# CMR T2* technique for segmental and global quantification of myocardial iron: multi-centre transfereability and healtcare impact evalaution

**DOI:** 10.1186/1532-429X-11-S1-O100

**Published:** 2009-01-28

**Authors:** Vincenzo Positano, Anna Ramazzotti, Antonella Meloni, Alessia Pepe, Giuseppe Rossi, Cristina Salvatori, Paolo Marcheschi, Maurizio Mangione, Luigi Natale, Eliana Cracolici, Gennaro Restaino, Gianluca Valeri, Antongiulio Luciani, Calogera Gerardi, Massimo Lombardi

**Affiliations:** 1 "G Monasterio" Foundation and Institute of Clinical Physiology, CNR, Pisa, Italy; 2 Policlinico "Gemelli", Roma, Italy; 3 Policlinico "Paolo Giaccone", Palermo, Italy; 4 Università Cattolica del Sacro Cuore, Campobasso, Italy; 5 Ospedali Riuniti di Ancona, Ancona, Italy; 6 Az. Osp. "Garibaldi", Catania, Italy; 7 Ospedali Civili Riuniti, Sciacca, (AG) Italy

**Keywords:** Cardiac Magnetic Resonance, Thalassemia, Chelation Therapy, Thalassemia Patient, Myocardial Iron

## Introduction

Iron induced cardiomiopathy is the main cause of mortality in thalassemic population. Thus, the improvement of chelation regimens, to reduce cardiac disease, has the highest priority. Efficient evaluation of cardiac iron status and careful epidemiologic assessment of thalassemic patients play an important role in this matter. T2* cardiac magnetic resonance imaging (CMR) is a unique technique to quantify myocardial iron overload and useful to tailor the chelation therapy. In particular, effective and reproducible assessment of myocardial iron loading using the multislice multiecho T2* approach for segmental and global myocardial iron distribution has been demonstrated within a single CMR site. Thalassemia major (TM) patients require lifelong myocardial iron load monitoring to assess the effectiveness of chelation therapies. Hence, it is highly desirable that CMR be performed near the patients' locations, and that the patients be able to safely move between different CMR centers.

## Purpose

Aim of this work is to build a reliable network of haematological and paediatric centers specializing in thalassemia care and MRI sites able to perform feasible and reproducible heart iron overload assessments for a consistent number of thalassemia patients in a standardized and robust manner.

## Materials and methods

In order to assess the transferability of the multislice multiecho T2* technique, heart multislice multiecho T2* sequence was installed on 1.5 T MRI scanners (GE Healthcare) at six different sites. Five healthy subjects at each site (n = 30) were scanned to verify the homogeneity of normal ranges (T2* lover limit of normal 20 ms). Then, five TM patients were scanned at the reference site and were rescanned locally (n = 25) within one month. T2* images were analysed using a previously validated software (HIPPO MIOT^®^).

After the assessment of CMR technique reproducibility, patients enrolling started in September 2006. A centralized data management system was made to share patient data between CMR and thalassemia sites. It allowed optimizing the TM patients care and favouring the creation of a clinical-instrumental database with data exchange facilities to develop diagnostic, prognostic and therapeutical evidence-based treatments for thalassemia patients. The study was approved by the local ethics committees and followed the principles outlined in the Declaration of Helsinki.

## Results

**Figure 1:**
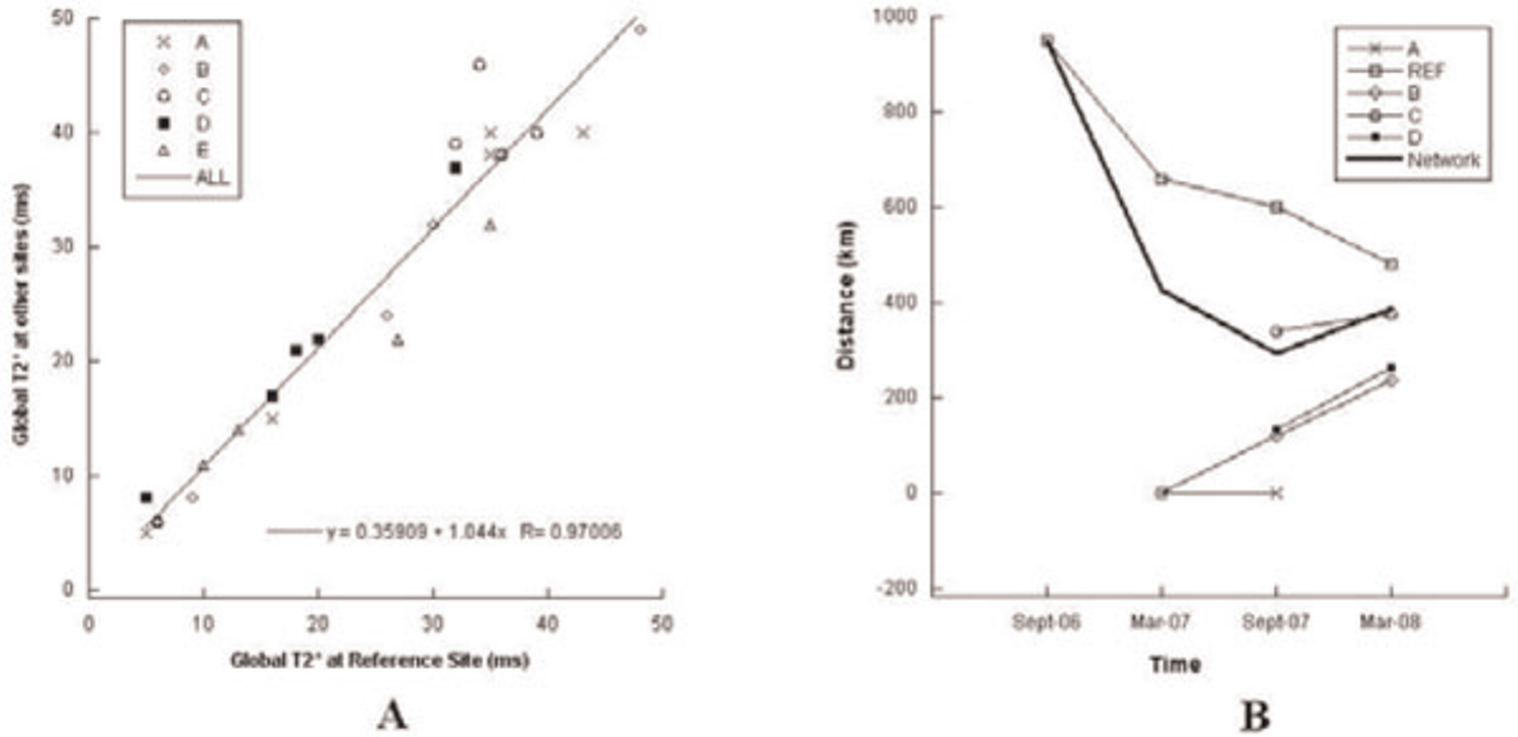
Figure 1

Global and segmental T2* values of healthy subjects showed inter-sites homogeneity. On TM patients, for global heart T2* values the correlation coefficient was 0.97, Coefficients of Variation (CoVs) ranged from 0.04 to 0.12 and Intraclass Coefficients (ICCs) ranged from 0.94 to 0.99. The mean CoV and ICC for segmental T2* distribution were 0.198 and 88, respectively. Figure 1A shows linear regression of global heart T2* values obtained from 25 (5 × 5) patients who were scanned at the reference site and locally at each of the other five sites on the same conditions.

Since the project's beginning, 695 thalassemia patients have been involved in the network. 630 patients (90%) successfully underwent CMR examination. Twenty patients (3%) refused CMR, mainly due to claustrophobia. The remaining patients (7%) have been scheduled for future examination. The mean distance from the patient home locations to the CMR site where the patients underwent the exams, which is considered an indicator of patient comfort, significantly decreased during the network's evolution. In Figure 1B, the average distances from the thalassemia centers which sent the patients for CMR examination are plotted versus time.

## Conclusion

The multislice multiecho T2* technique is transferable among scanners with good reproducibility. The network seems to be a robust and scalable system in which T2* CMR-based cardiac iron overload assessment is available, accessible and reachable for a significant and increasing number of thalassemia patients, reducing the mean distance from the patients' locations to the CMR sites.

